# Local ice cryotherapy decreases synovial interleukin 6, interleukin 1β, vascular endothelial growth factor, prostaglandin-E2, and nuclear factor kappa B p65 in human knee arthritis: a controlled study

**DOI:** 10.1186/s13075-019-1965-0

**Published:** 2019-07-30

**Authors:** X. Guillot, N. Tordi, C. Laheurte, L. Pazart, C. Prati, P. Saas, D. Wendling

**Affiliations:** 1 Department of Rheumatology, Felix Guyon University Hospital, Saint-Denis, Reunion France; 2 PEPITE EA4267, FHU INCREASE, Bourgogne-Franche-Comté University, Besançon, France; 3 Department of Rheumatology, Besançon university hospital, Besançon, France; 4 INSERM U1098, Biomonitoring Platform, EFS, Besançon University Hospital, Besançon, France; 5 CIC IT, INSERM Center CIT 808, Besançon University Hospital, Besançon, France; 6 EA 4266, Bourgogne-Franche-Comté University, Besançon, France

**Keywords:** Local cryotherapy, Knee arthritis, Cytokines, PG-E2, NF-kB

## Abstract

**Background:**

The aim of this study was to assess the anti-inflammatory effects of local cryotherapy in human non-septic knee arthritis.

**Methods:**

In the phase I of the study, patients were randomized to receive either ice (30 min; *N* = 16) or cold CO_2_ (2 min; *N* = 16) applied twice during 1 day at an 8-h interval on the arthritic knee. In phase II, 16 other ice-treated arthritic knees according to the same protocol were compared to the contralateral non-treated arthritic knees (*N* = 16). The synovial fluid was analyzed just before the first cold application, then 24 h later. IL-6, IL-1β, TNF-α, IL-17A, VEGF, NF-kB-p65 protein, and PG-E2 levels were measured in the synovial fluid and compared before/after the two cold applications.

**Results:**

Forty-seven patients were included (17 gouts, 11 calcium pyrophosphate deposition diseases, 13 rheumatoid arthritides, 6 spondyloarthritides). Local ice cryotherapy significantly reduced the IL-6, IL-1β, VEGF, NF-kB-p65, and PG-E2 synovial levels, especially in the microcrystal-induced arthritis subgroup, while only phosphorylated NF-kB-p65 significantly decreased in rheumatoid arthritis and spondyloarthritis patients. Cold CO_2_ only reduced the synovial VEGF levels. In the phase II of the study, the synovial PG-E2 was significantly reduced in ice-treated knees, while it significantly increased in the corresponding contralateral non-treated arthritic knees, with a significant inter-class effect size (mean difference − 1329 [− 2232; − 426] pg/mL; *N* = 12).

**Conclusions:**

These results suggest that local ice cryotherapy reduces IL-6, IL-1β, and VEGF synovial protein levels, mainly in microcrystal-induced arthritis, and potentially through NF-kB and PG-E2-dependent mechanisms.

**Trial registration:**

Clinicaltrials.gov, NCT03850392—registered February 20, 2019—retrospectively registered

**Electronic supplementary material:**

The online version of this article (10.1186/s13075-019-1965-0) contains supplementary material, which is available to authorized users.

## Background

Cryotherapy (applied locally or to the whole body) has been widely and empirically used in inflammatory rheumatic diseases, as an adjunct therapy, with a low level of evidence [1]. Local cryotherapy consists in the local application of ice, cold packs [2] (inducing a progressive and prolonged cooling), or cold gases (also called cryostimulation—inducing more ample and brutal temperature drops but for shorter durations). This second type of technique induces a physiological response called “thermal shock,” inducing additional vaso-active effects (a brutal vaso-constriction followed by a vaso-dilatation, called “hunting reaction” through a sympatho-adrenal nervous system involvement), endogenous norepinephrine, and cortisol secretions [3]. Whole-body cryotherapy is also mostly based on cold gas application and cryostimulation [4], notably in cryogenic chambers, inducing a more global cooling [5]. Whole-body cryotherapy could exert beneficial systemic analgesic [6], myo-relaxing [7], anti-inflammatory [8], and anti-oxidative [9] effects in a wide panel of musculo-squeletal disorders. However, the cryotherapy protocols (physical agents, temperature, duration) are not standardized, and methodological issues, such as the difficulty to conceive placebo groups and frequent concomitant therapeutics (anti-inflammatory drugs, kinesitherapy, physical exercise), prevent the existing studies from being fully conclusive. We could show that local ice or cold CO_2_ applied twice over 1 day significantly decreased the power Doppler semi-quantitative score after 24 h in 30 patients suffering from non-septic knee arthritis [10]. However, the molecular pathways involved in this anti-inflammatory effect remain widely unknown. In a rat adjuvant-induced arthritis model, we could show that local cryotherapy (ice (30 min) or cold gas (2 min)) applied twice a day for 14 consecutive days to the arthritic hind paws significantly improved the arthritis score and reduced the IL-6 and IL-17A local and plasmatic levels, both at the gene and protein levels, compared to non-treated controls, with no effect on the TNF-α pathway [11]. In this model, ice was more effective and better tolerated compared to cold gas. In humans, some non-controlled studies suggest that local and whole-body cryotherapy might reduce cytokine plasma levels, with conflicting results and numerous biases such as concomitant anti-inflammatory drugs [12–15]. These anti-cytokine effects might be related to cryotherapy-induced tissue mild hypothermia (30–34 °C), which showed anti-inflammatory properties through nuclear factor kappa B (NF-kB)-dependent cytokine gene transcription inhibition [16], and by repressing the pivotal pro-inflammatory enzyme pathways such as cyclo-oxygenase 2 (COX-2) [17], collagenases [18], and pro-angiogenic factors like vascular endothelial growth factor (VEGF) [19]. Moreover, one single 30-min local ice application was shown to reduce knee intra-joint temperature to 30 °C for 2 h in rheumatoid arthritis (RA) patients [20].

The aim of this study was to further elucidate the molecular pathways involved in local cryotherapy’s anti-inflammatory effects in patients suffering from non-septic knee arthritis. Therefore, interleukin 6 (IL-6), IL-1β, IL-17A, tumor necrosis factor alpha (TNF-α), prostaglandin E2 (PG-E2), NF-kB-p65, and phosphorylated NF-kB-p65 (NF-kB-P65-P) levels were measured in the synovial fluid before and 24 h after the first of two cold applications in cryotherapy-treated knees and also in contralateral non-treated arthritic knees when possible. We also aimed at comparing two techniques (ice and cold CO_2_) applied twice within 1 day. The effect on pain visual analog scale (pain VAS) and the tolerance were also considered.

## Methods

### Patient inclusion

Patients hospitalized in the Rheumatology Department at the Besançon University Hospital in France and suffering from non-septic knee arthritis (RA according to the ACR-EULAR criteria, spondyloarthritis (SpA) according to the ASAS criteria, gout, or calcium pyrophosphate deposition disease (CPDD)—diagnosed by microscopic microcrystal assessment in synovial fluid) were included consecutively after they signed the informed consent. The protocol was declared and approved by the local ethics committee (clinicaltrials.gov: NCT03850392, Comité de Protection des Personnes - Est II: 12-664), and all research was performed in accordance with relevant guidelines and regulations. Patients suffering from septic arthritides and knee osteoarthritis were excluded. The patients had received no biologic treatment nor conventional DMARD for 6 months preceding the inclusion. Corticosteroids, colchicine, and NSAIDs were stopped for at least 24 h prior to inclusion.

### Study design

In the first phase of the study, the included patients were then randomized (1:1) to receive either local ice (Thermogel®, Artsana, Grandate, Italy—30-min application; *N* = 16) or hyperbaric cold CO_2_ at − 78 °C (Cryo+®, Cryonic, Salins-les-Bains, France—2 min; *N* = 16). Each patient received two applications of the randomly chosen technique at an 8-h interval (9 a.m. and 5 p.m). The skin temperature was monitored on the treated knee using MLT409/A Skin Temperature Probe® and ML309 Thermistor Pod® (ADInstruments, Dunedin, NZ). Just before the first cold application, at 9 a.m., and 24 h later (day 1 at 9 a.m), an arthrocentesis was performed. Standard analyses were performed on the synovial fluid (bacteriology, cytology, and microcrystal microscopic assessment). Furthermore, a part of the synovial fluid was centrifuged then frozen at − 80 °C. For the second arthrocentesis, after the synovial fluid was gathered for the same analyses, an intra-joint corticosteroid injection (triamcinolone, HEXATRIONE®, Ethypharm, Saint-Cloud, France) was performed before removing the needle. These synovial fluid samples were used to perform the present part of the study, which was overall powered to evaluate the IL-6 level variations in the synovial fluid before/after two cold applications. After all the patients were included, synovial fluid IL-6, IL-17A, IL-1β, TNF-α, VEGF (multiplex flow cytometry, CBA® BD Bioscience, Franklin Lakes, NJ, USA), PG-E2 (ELISA, KGE004B®, Bio-Techne, Minneapolis, MN, USA), and NF-KB-P65/NF-kB-p65-P (ELISA, 85-86083-11®, Thermofisher, Waltham, MA, USA) levels were measured.

**Fig. 1:**
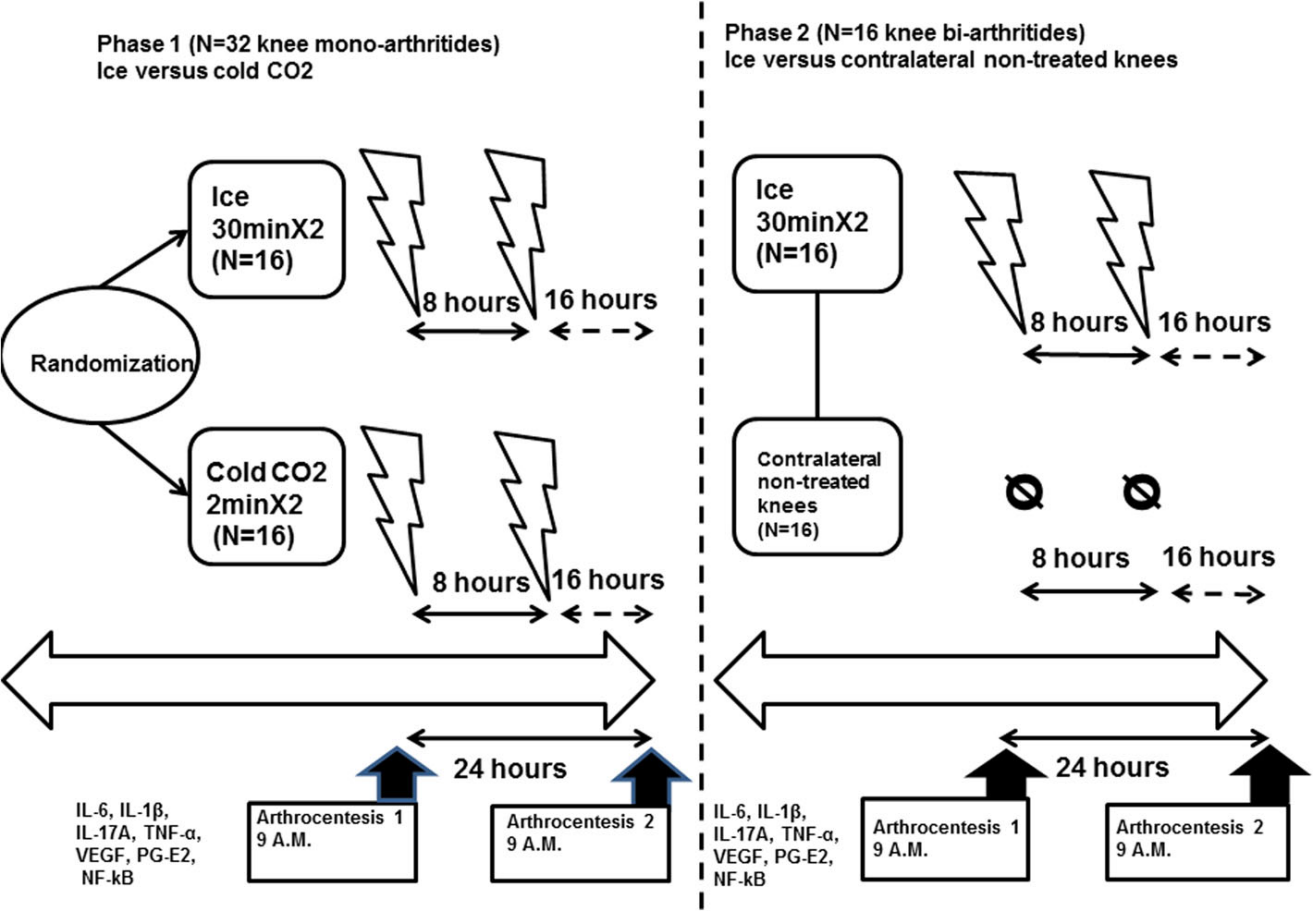
Study design. In the first phase of the study, patients were randomized to receive either ice (30 min; *N* = 16) or cold CO_2_ (2 min; *N* = 16) twice at an 8-h interval. In the second phase, 16 patients with arthritides of both knees were included. One of the knees was treated with local ice (*N* = 16) while the contralateral arthritic knees were used as paired controls (*N* = 16) for the synovial fluid analyses. One of these 16 patients had been previously included in the first phase of the study (ice group). Therefore, a total of 47 patients were included. “**⍉**” indicates no cryotherapy treatment

In the second phase of the study, we only included patients suffering from arthritides of both knees and treated them with local ice only, according to the protocol described above (*N* = 15 + 1 patient with knee bi-arthritis previously included in the first phase of the study in the ice-treated group). The same protocol was applied to contralateral non-treated knees except cryotherapy treatment. Therefore, the synovial fluid was gathered and analyzed at the same evaluation times compared to the treated knees, so these contralateral arthritic knees were used as paired controls for cytokine and enzyme assays (*N* = 16). The study design is summarized in Fig. 1**.**

### Statistical analyses

The sample size was calculated in order to detect a significant variation in IL-6 synovial protein levels before/after 2 cold applications. 15.78 (*N* = 16) patients per group were necessary to detect a difference of 2325 pg/mL in IL-6 protein level with a power of 95% and a *p* value of 0.05, according to published results of IL-6 assays in knee synovial fluid [21]. Therefore, 2 groups of 16 patients were included in the first randomized phase of the study (ice versus cold CO_2_), then 16 patients with knee bi-arthritis were required for the second phase of the study (ice-treated versus contralateral knee, *N* = 15 + 1 patient already enrolled in the first phase). For these reasons, a total of 47 patients were included. Paired Wilcoxon-Mann-Whitney tests were performed in order to compare the mean cytokine and enzyme levels before/after treatment. Subgroup analyses were also planned (according to the treatment modalities (ice or cold CO_2_) and to the type of rheumatic disease (microcrystal-induced arthritides—pooled gout and CPDD patients—versus non-microcrystal-induced diseases—pooled RA and SpA patients). Furthermore, an inter-class effect size (weighted mean differences with 95% CI) for cytokine levels (before/after treatment) was calculated between ice-treated knees and the corresponding contralateral non-treated knees using R® software (rmeta® and meta® packages). Correlation tests were also performed using Pearson’s coefficients in order to assess the parameters associated with cytokine level variations (before/after treatment). The statistical analyses were performed using R® and Graphpad® softwares.

## Results

Forty-seven patients were included, as initially planned (Fig. 1). No dropout nor side effect was observed. The patient characteristics are summarized in Table 1**.**

**Table 1:** Patient characteristics

	Ice + cold CO_2_ (*N* = 47)	Ice (*N* = 31)	Ice (randomized phase, *N* = 16)	Cold CO_2_ (*N* = 16)	*p* value (ice versus CO_2_, randomized phase, *N* = 16 versus 16)	*p* value (ice versus CO_2_, *N* = 31 versus 16)
Diagnosis	RA (*N* = 13), SpA (*N* = 6), gout (*N* = 17), CPDD (*N* = 11)	RA (*N* = 10), SpA (*N* = 2), gout (*N* = 9), CPDD (*N* = 10)	RA (*N* = 3), SpA (*N* = 1), gout (*N* = 6), CPDD (*N* = 6)	RA (*N* = 3), SpA (*N* = 4), gout (*N* = 8), CPDD (*N* = 1)	0.43	0.069
Age (years)	60.1 [54.7–65.6]	65.8 [59.6–71.9]	65.9 [56.7–75.2]	49.1 [39.9–58.4]	0.007**	0.002**
Sex	M:25; F:22	M:14; F:17	M:11; F:5	M:11; F:5	1	0.22
BMI (kg/m^2^)	25.7 [24.3–27.1] (NA:1)	26.1 [24.2–27.9] (NA:1)	26.3 [23.4–29.3] (NA:1)	24.9 [22.6–27.3]	0.54	0.51
CRP (mg/L)	110.5 [85.5–135.4]	122.1 [87.9–156.2]	131.6 [83.9–179.3]	88 [54.5–121.5]	0.19	0.4
First arthrocentesis volume (mL)	15.7 [13.5–18]	14.2 [12.4–16]	15.4 [12.4–18.3]	18.7 [13–24.4]	0.4	0.13
Synovial leukocyte count (G/L)	18.242 [11.158–25.326] (NA:9)	17.277 [8.273–26.281] (NA:5)	25.025 [6.702–43.348] (NA:4)	20.333 [8.134–32.532] (NA:4)	0.73	0.67

Results are presented as means ± 95% confidence intervals. Means were compared using Wilcoxon-Mann-Whitney tests, while percentages were compared using Fisher tests

*BMI* body mass index, *CRP* C-reactive protein, *NA* not assessed

*p < 0.05; **p < 0.01; ***p < 0.001

The mean skin temperatures observed in each treatment group and at each evaluation time during the randomized phase were published elsewhere [10], and the mean maximal temperature drops during the first cryotherapy application were − 17.9 ± 0.85 °C (SEM) in ice-treated knees (*N* = 31) and significantly lower − 24.6 °C ± 0.93 °C in CO_2_-treated knees (*N* = 16)—***p < 0.0001.

The mean pain VAS (0–10) was significantly lower after two cold applications (i.e., 24 h after the first cold application) in both treatment groups: 3.3 ± 0.41 versus 5.6 ± 0.52, ***p = 0.00002, in ice-treated patients (*N* = 31) and 3.3 ± 0.6 versus 5.6 ± 0.69, **p = 0.002, in CO_2_-treated patients (*N* = 16).

### Phase I: Randomized comparison of ice- (*N* = 16) versus cold CO_2_- (*N* = 16) treated patients (Fig. 2)

**Fig. 2:**
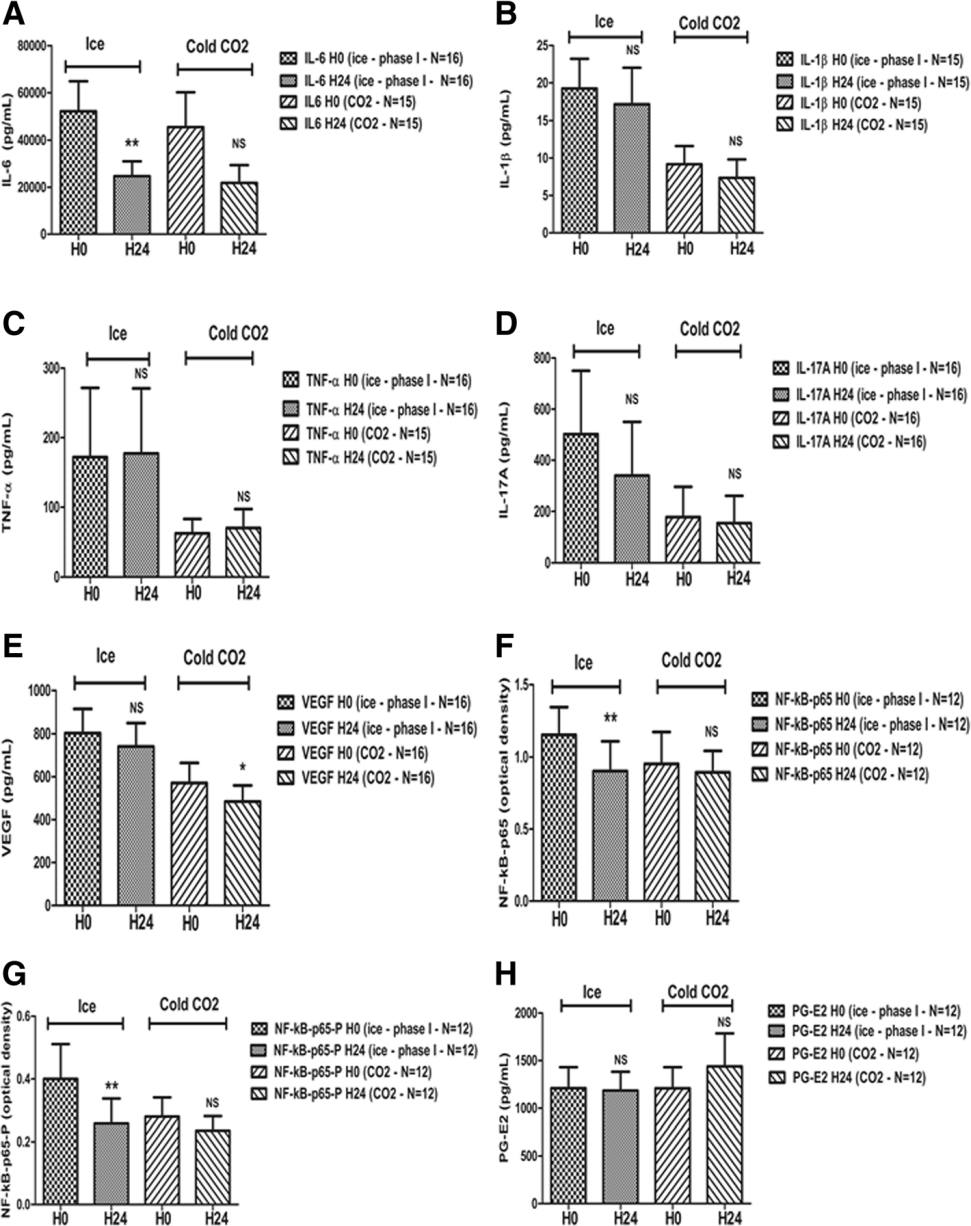
Pro-inflammatory mediator evolution after two cold applications in the synovial fluid of treated knees: randomized comparison of ice- (*N* = 16) versus cold CO_2_- (*N* = 16) treated patients (phase I). Thirty-two patients were first randomized to receive either local ice (30 min twice; *N* = 16) or cold CO_2_ (2 min twice; *N* = 16) on an arthritic knee. The synovial fluid was analyzed before and after treatment as previously described. Synovial IL-6 (**a**), IL-1β (**b**), TNF-α (**c**), IL-17A (**d**), and VEGF (**e**) assessed by multiplex flow cytometry (CBA® BD Bioscience, Franklin Lakes, NJ, USA); NF-KB-p65 (**f**)/NF-kB-p65-P (**g**) assessed by ELISA (85-86083-11®, Thermofisher, Waltham, MA, USA); and PG-E2 (**h**) assessed by ELISA (KGE004B®, Bio-Techne, Minneapolis, MN, USA) levels were all measured at 9 a.m. (just before the first cold application, then 24 h later). Data are presented as means ± SEM. Paired Wilcoxon-Mann-Whitney tests were performed. *p < 0.05, **p < 0.01, ***p < 0.001. H0: first evaluation (9 a.m., before the first cold application); H24: second evaluation (9 a.m., 24 h later, after the two cold applications). Missing values were due to the fact that some cytokines and mediators could not be detected—or with out-of-range values—in some synovial fluid samples before and/or after cold application (IL-6: *N* = 1 in the CO_2_ group at H24; IL-1β: *N* = 1 at H24 in the ice group and *N* = 1 both at H0 and H24 in the CO_2_ group; TNF-α: *N* = 1 both at H0 and H24 in the CO_2_ group; IL-17A: *N* = 0; VEGF: *N* = 0; NFkB-p65: *N* = 4 in the CO_2_ (1 at H0, 1 at H24, 1 at both H0 and H24) and ice (2 at H0, 2 at H24) groups; NFkB-p65P: *N* = 4 in the CO_2_ (1 at H0, 1 at H24, 1 at both H0 and H24) and ice (2 at H0, 2 at H24) groups; PG-E2: *N* = 4 in the CO_2_ (1 at H0, 1 at H24, 1 at both H0 and H24) and ice (2 at H0, 2 at H24) groups)

In the first randomized phase of the study, synovial IL-6 significantly decreased in the first 16 ice-treated patients, while it did not vary significantly in the CO_2_-treated patients (Fig. 2a). NF-kB-p65 also significantly decreased in the ice-treated group while it did not change significantly in CO_2_-treated patients (Fig. 2f). NF-kB-p65-P showed a similar variation pattern (Fig. 2g). Conversely, VEGF significantly decreased in CO_2_-treated patients while the decrease was not significant in ice-treated patients (Fig. 2e).

### Phase II: Contralateral non-treated knees (*N* = 16; Fig. 3)

**Fig. 3:**
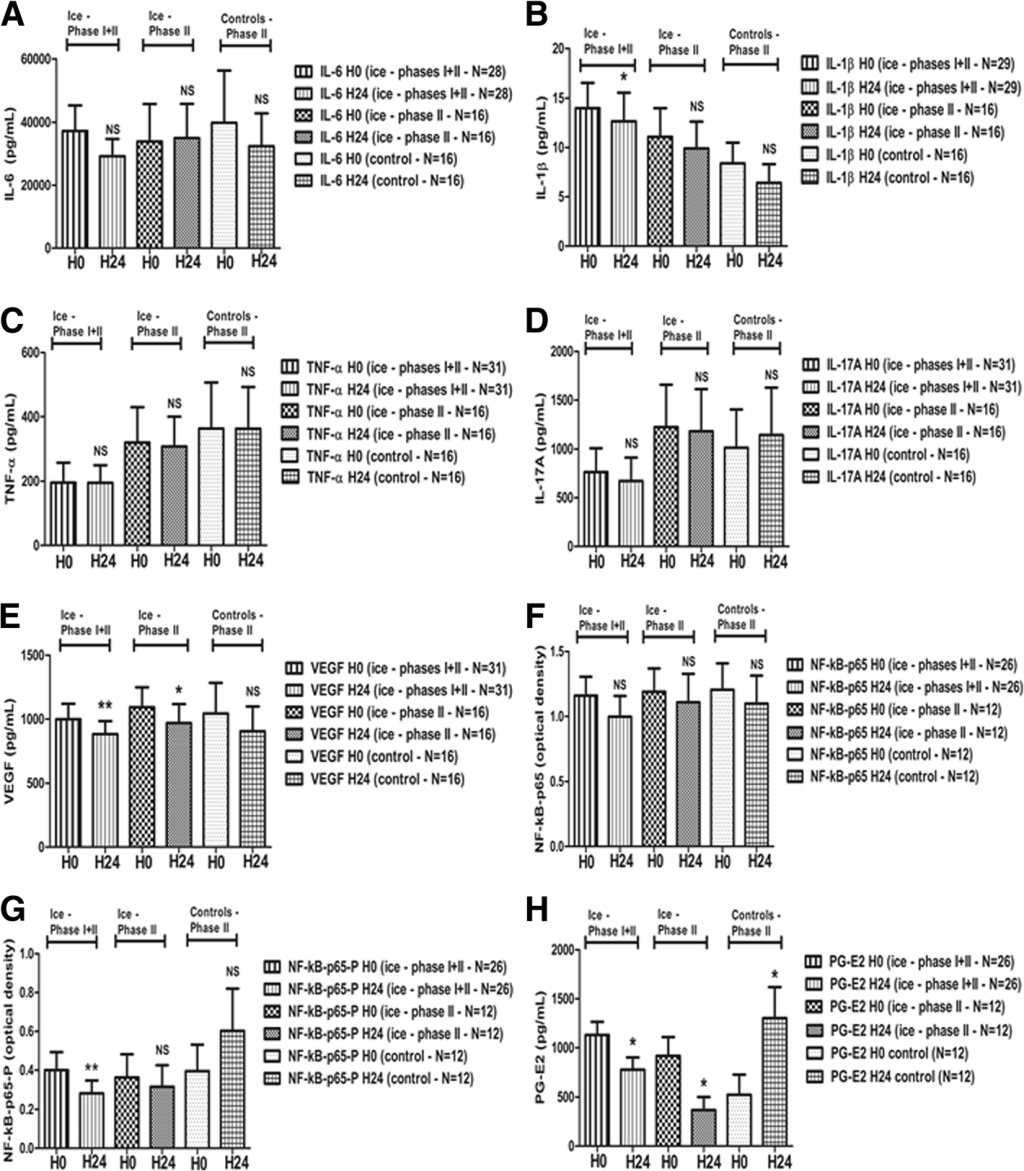
**a**–**h** Pro-inflammatory mediator evolution after two cold applications in the synovial fluid of ice-treated knees (*N* = 16) compared to contralateral non-treated arthritic knees (*N* = 16) in knee bi-arthritic patients (phase II). Sixteen patients with knee bi-arthritis were then included (phase II). One knee was treated with ice according to the study protocol (*N* = 16). The contralateral knees were used as paired controls (*N* = 16—on the right part of the graphs). The synovial fluid was analyzed before and after treatment as previously described, on both knees for each patient. The results of synovial fluid analyses of all the ice-treated patients (phase I + II; *N* = 31) are also shown on the left part of the graphs, for comparison. Data are presented as means ± SEM. Wilcoxon-Mann-Whitney tests were performed. *p < 0.05, **p < 0.01, ***p < 0.001. H0: first evaluation (9 a.m., before the first cold application); H24: second evaluation (9 a.m., 24 h later, after the two cold applications); controls: contralateral non-treated arthritic knees. Missing values were due to the fact that some cytokines and mediators could not be detected—or with out-of-range values—in some synovial fluid samples before and/or after cold applications (IL-6: *N* = 3 in ice-treated phase I + phase II patients (1 at H0, 2 at both H0 and H24), *N* = 0 in ice-treated phase II and contralateral non-treated knees; IL-1β: *N* = 2 in ice-treated phase I + phase II patients (1 at H0, 1 at H24), *N* = 0 in ice-treated phase II and contralateral non-treated knees; TNF-α: *N* = 0; IL-17A: *N* = 0; VEGF: *N* = 0; NFkB-p65: *N* = 5 in ice-treated phase I + phase II patients (2 at H0, 3 at H24), *N* = 0 in ice-treated phase II, *N* = 4 in contralateral non-treated knees (2 at H0, 1 at H24, 1 at both H0 and H24); NFkB-p65P: *N* = 5 in ice-treated phase I + phase II patients (2 at H0, 3 at H24), *N* = 0 in ice-treated phase II, *N* = 4 in contralateral non-treated knees (2 at H0, 1 at H24, 1 at both H0 and H24); PG-E2: *N* = 5 in ice-treated phase I + phase II patients (2 at H0, 3 at H24), *N* = 0 in ice-treated phase II, *N* = 4 in contralateral non-treated knees (2 at H0, 1 at H24, 1 at both H0 and H24))

In contralateral non-treated knees from 16 ice-treated patients, the mean PG-E2 levels significantly increased while they significantly decreased in the corresponding 16 ice-treated knees (Fig. 3h). The inter-class effect size was significant (weighted mean difference − 1329, 95% CI [− 2231.7; − 425.64] pg/mL, *N* = 12 bi-arthritic patients; Additional file 1: Figure S1).

The other cytokines and enzymes did not vary significantly in contralateral non-treated knees (Figs. 2 and 3, right columns).

When considering all ice-treated patients (phase I + II, *N* = 31), IL-1β significantly decreased (Fig. 3b). We also observed significant decreases in VEGF (Fig. 3e), NF-kB-p65-P (Fig. 3g), and PG-E2 (Fig. 3h) levels (Fig. 3: two left columns).

**Fig. 4:**
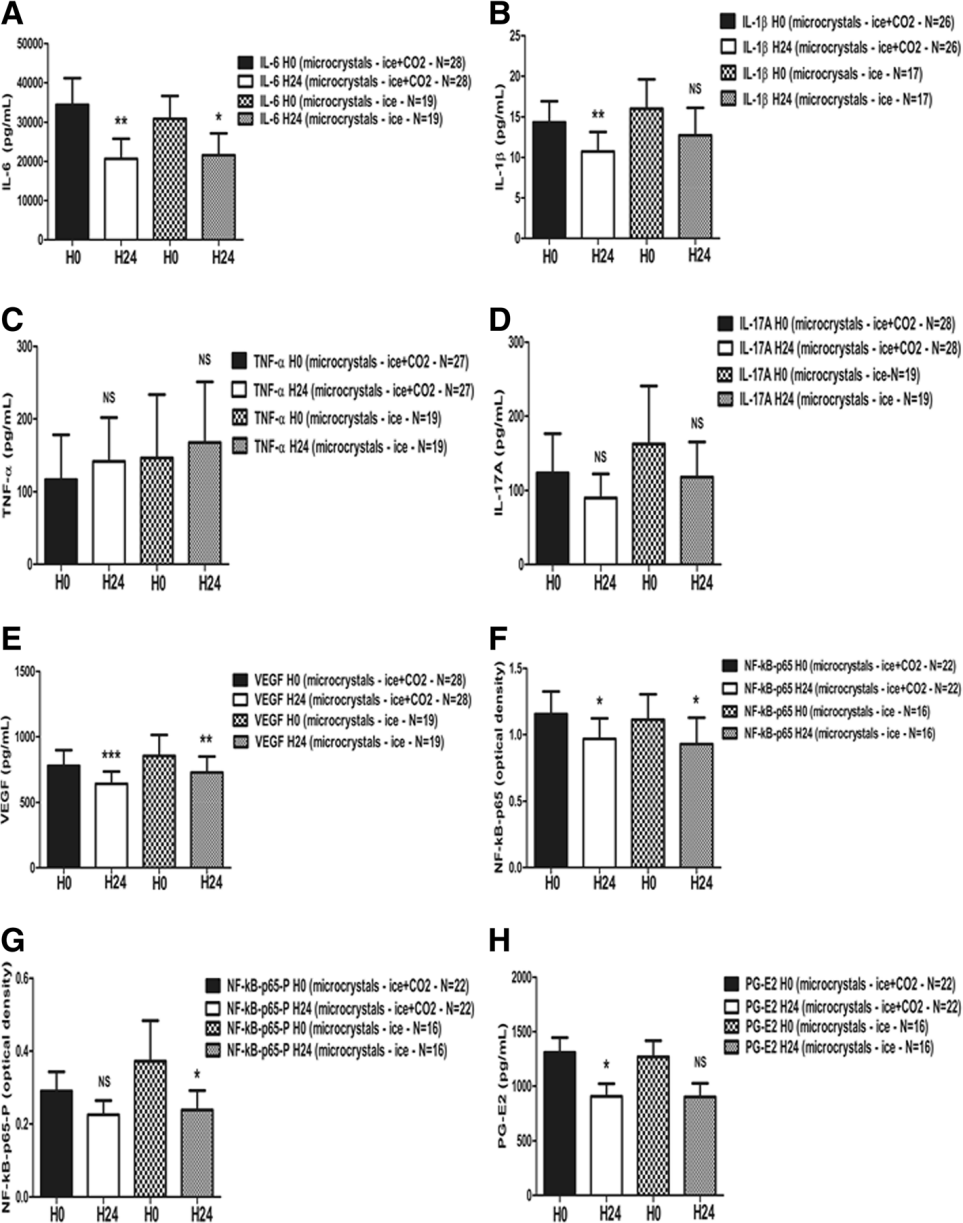
**a**–**h** Pro-inflammatory mediator evolution after two cold applications in the synovial fluid in the microcrystal-induced arthritis subgroup (*N* = 28; ice: *N* = 19). The synovial fluid of all cryotherapy-treated patients suffering from microcrystal-induced arthritis (*N* = 27 including 18 ice-treated patients) was analyzed just before the first cold application, then 24 h later (after two cold application). Data are presented as means ± SEM. Paired Wilcoxon-Mann-Whitney tests were performed. *p < 0.05, **p < 0.01, ***p < 0.001. H0: first evaluation (9 a.m., before the first cold application); H24: second evaluation (9 a.m., 24 h later, after the two cold applications); microcrystals: patients suffering from microcrystal-induced knee arthritis. Missing values were due to the fact that some cytokines and mediators could not be detected—or with out-of-range values—in some synovial fluid samples before and/or after cold applications (IL-6: *N* = 0; IL-1β: *N* = 2 in ice-treated patients (1 at H0, 1 at H24); TNF-α: *N* = 1 (1 CO_2_-treated patient both at H0 and H24); IL-17A: *N* = 0; VEGF: *N* = 0; NFkB-p65: *N* = 6 in ice + CO_2_-treated patients (3 at H0, 2 at H24, 1 CO_2_-treated patient at both H0 and H24), *N* = 3 in ice-treated patient (2 at H0, 1 at H24); NFkB-p65P: *N* = 6 in ice + CO_2_-treated patients (3 at H0, 2 at H24, 1 CO_2_-treated patient at both H0 and H24), *N* = 3 in ice-treated patient (2 at H0, 1 at H24); PG-E2: *N* = 6 in ice + CO_2_-treated patients (3 at H0, 2 at H24, 1 CO_2_-treated patient at both H0 and H24), *N* = 3 in ice-treated patient (2 at H0, 1 at H24))

When considering microcrystal-induced arthritides separately (*N* = 28), the decreases in IL-6 (Fig. 4a), IL-1β (Fig. 4b), and VEGF (Fig. 4e) were more pronounced while the variations in IL-6 (Additional file 2: Figure S2A), IL-1β (Additional file 2: Figure S2B), and VEGF (Additional file 2: Figure S2E) levels were non-significant in RA and SpA patients. Furthermore, the NF-kB-p65 (Fig. 4f) and PG-E2 (Fig. 4h) decreases became significant in microcrystal-induced arthritides, while PG-E2 did not vary significantly in RA and SpA patients (Additional file 2: Figure S2H). By contrast, NF-kB-p65-P decreased non-significantly in microcrystal-induced arthritides (Fig. 4g), while it significantly decreased in RA and SpA patients (Additional file 2: Figure S2G).

When focusing on the ice-treated microcrystal-induced arthritis patients (*N* = 19), the decrease in synovial IL-6 protein levels became statistically significant (Fig. 4a), while this decrease was non-significant in RA and SpA patients (Additional file 2: Figure S2A). This was also the case for NF-kB-p65 (Fig. 4f and Additional file 2: Figure S2F). As concerns IL-1β, the decrease was non-significant both in ice-treated microcrystal-induced arthritides (Fig. 4b) and in RA and SpA patients (Additional file 2: Figure S2B). This was also the case for VEGF (Fig. 4e, Additional file 2: Figure S2E). For NF-kB-p65-P, we observed a significant decrease in ice-treated microcrystal-induced arthritis patients (Fig. 4g) while the difference was not significant in RA and SpA ice-treated patients (Additional file 2: Figure S2G). For PG-E2, the decreases became non-significant when considering microcrystal-induced arthritis patients (Fig. 4h, Additional file 2: Figure S2H).

By contrast, TNF-α and IL-17A did not vary in any treatment group nor in any phase of the study (Figs. 2c, d; 3c, d; 4c, d; Additional file 2: Figure S2C, 2D).

### Correlations

The synovial NF-kB-p65 decrease correlated significantly with the synovial IL-6 decrease (Additional file 3: Figure S3A) and the synovial VEGF decrease (Additional file 3: Figure S3B). The synovial NF-kB-p65-P decrease also correlated with the synovial VEGF decrease (Additional file 3: Figure S3C). Furthermore, the synovial NF-kB-p65 decrease correlated significantly with the maximal skin temperature drop induced by local cryotherapy (Additional file 3: Figure S3D), and the synovial VEGF decrease correlated with the maximal skin temperature drop induced by local cryotherapy (Additional file 3: Figure S3E).

## Discussion

In this study, we demonstrated for the first time that local cryotherapy applied twice inhibits pivotal pro-inflammatory cytokine and enzyme pathways in the synovial fluid of non-septic arthritic knees. First, in the randomized phase I of the study (1:1; *N* = 32), local ice induced significant decreases in IL-6, NF-kB-p65, and NF-kB-p65-P protein levels, while cold CO_2_ only reduced the VEGF levels (Fig. 2). Secondly, in the phase II of the study, when considering patients with arthritis of both knees, the PG-E2 synovial levels significantly decreased in ice-treated knees (*N* = 12), while they significantly increased in contralateral non-treated knees (*N* = 12; Fig. 3h), with a significant inter-class effect size (Additional file 1: Figure S1). Thirdly, when pooling the results from all ice-treated patients from phase I + phase II (*N* = 31), the IL-1β, VEGF, NF-kB-p65-P, and PG-E2 levels were significantly lower after two cold applications (Fig. 3). When considering only microcrystal-induced arthritides, the IL-6, and NF-kB-p65 protein levels were also significantly lower in these ice-treated patients (*N* = 19; Fig. 4). Fourthly, in the subgroup of patients suffering from RA and SpA (*N* = 19), local cryotherapy only reduced the NF-kB-p65-P protein levels (ice + CO_2_; *N* = 16; Additional file 2: Figure S2G). Fifthly and overall, local cryotherapy had no effect on the TNF-α nor IL-17A synovial levels.

In the murine model of adjuvant-induced arthritis, we had previously shown that subchronic local cryotherapy—especially local ice—applied twice a day for 14 consecutive days significantly reduced local and systemic IL-6, both at the gene and protein levels, compared to non-treated arthritic controls, with no effect on local and systemic TNF-α [11]. In this animal study, local cryotherapy also markedly inhibited local and systemic IL-17A levels, which was not the case in the present clinical trial. This difference is probably due to the short treatment regimen (two applications) we used in our arthritic patients. Indeed, in an arthritic rat patella culture model, punctual tissue hypothermia (30 °C for 2 h—in order to mimic the tissue mild hypothermia induced by a single ice application on an arthritic knee [20]) only repressed the IL-6 protein levels in culture supernatants, with no effect on the IL-17A protein levels [11]. This might be due to the fact that the inhibitory effects of local cryotherapy on IL-6—potentially through NF-kB-dependent gene transcription inhibition [16]—could be more precocious than those on IL-17A, as IL-6 is required to induce subsequent CD4+ T lymphocyte Th-17 differentiation and IL-17A production [22, 23].

As for the underlying molecular mechanisms, the cytokine protein decreases we observed after two local cryotherapy applications could be related to a NF-kB-dependent cytokine gene transcription inhibition [24] and to a temperature-dependent inhibition of the COX-2/PG-E2 pathway [17]. Indeed, mild hypothermia was shown to inhibit important enzyme pathways involved in joint inflammation and destruction such as collagenases [18] and metalloproteinases [25, 26]. Mild hypothermia had also been shown to reduce VEGF protein levels in retinal cell culture experiments [19]. These hypotheses are corroborated by our results, as we observed significant positive correlations between NF-kB-p65 on the one hand and IL-6 as well as VEGF protein level variations on the other hand (Additional file 3: Figure S3A, S3B, S3C). Furthermore, we observed significant correlations between the maximal skin temperature drop induced by local cryotherapy and NF-kB-p65 as well as VEGF protein variations in the synovial fluid after treatment (Additional file 3: Figure S3D, S3E). Surprisingly, these two last correlations were negative, potentially indicating that tissue hypothermia, when too intense, might be less efficient in reducing inflammation, or even become pro-inflammatory beyond a certain intensity or duration threshold.

Overall, ice application was more efficient than cold CO_2_ in reducing synovial inflammation in our study (Fig. 2). In our animal study, local ice was also more efficient in reducing joint inflammation compared to cold gas, and better tolerated [11]. This might be explained by the fact that local ice (applied for 30 min) could induce a more in-depth and longer-term tissue hypothermia, and therefore inhibit more efficiently the NF-kB and PG-E2 pathways, compared to cold CO_2_, applied for only 2 min, inducing a more brutal and ample skin temperature drop but also a more superficial and brief tissue hypothermia. We cannot exclude that the sample size might have been insufficient to demonstrate the effects of cold CO_2_ on some cytokines and enzymes, as it was only calculated to show a significant decrease in synovial IL-6 (*N* = 16). By contrast, a total of 31 patients were treated with ice, as this modality was also used in the phase II of the study (due to its superior efficacy in phase I). Conversely, in phase I, cold CO_2_ seemed to be more efficient than ice in reducing the synovial VEGF levels, potentially due to the more pronounced vaso-active effects compared to ice (“thermal shock” and vaso-constriction).

We observed that the decrease in synovial IL-6 was significant in ice-treated patients during the randomized phase I of the study (*N* = 16; Fig. 2a) but became non-significant in phase II (*N* = 16) and when pooling all ice-treated patients (*N* = 31; Fig. 3a). However, when considering the subgroup of microcrystal-induced arthritides, IL-6 significantly decreased in ice-treated patients (*N* = 19; Fig. 4a) but also in the 3 microcrystal-induced arthritis patients of phase II (data not shown). This relative underrepresentation of microcrystal-induced arthritides (3 out of 12) compared to RA and SpA might be the reason why we could not demonstrate a significant effect size on the synovial IL-6 levels compared to contralateral non-treated knees in the phase II of the study. Overall, these results suggest that local ice cryotherapy might be more efficient in reducing the synovial IL-6 levels in microcrystal-induced arthritis compared to RA and SpA. No data in the literature support this hypothesis, as local cryotherapy was poorly studied in microcrystal-induced arthritis so far [27].

Local ice induced a marked decrease in the synovial PG-E2 levels, especially in the phase II of the study, versus contralateral non-treated knees (Fig. 3h, Additional file 1: Figure S1). This result suggests that local ice might exert anti-cyclo-oxygenase-2 (anti-COX-2)-like effects. The profile of the anti-cytokine effects we observed (IL-6 decrease, no effect on TNF-α) also suggests that the effects of local ice might be anti-COX-2-like rather than corticosteroid-like [28]. The COX-2 levels could only be measured by ELISA in the synovial fluid of two patients in our study before and after local cryotherapy (one ice-treated and one CO_2_-treated), and they decreased in both cases (data not shown).

The cooling protocols we used and the skin temperature drops we observed were in line with those described in the literature [1]. Local ice applied for 30 min on RA patient knees was shown to reduce the intra-joint temperature to 30 °C for 2 h—therefore inducing tissue mild hypothermia [20]. As for cold CO_2_, we followed the manufacturer’s recommendations (2-min applications twice a day), and the 4 °C skin temperature target (inducing a “thermal shock”) was reached in all treated patients [3].

As for longer-term cold application on arthritic joints, the optimal protocol could be twice a day for 14 consecutive days, which showed beneficial effects on pain and disease activity in RA patients [1], This subchronic application protocol, when applied in the murine model of adjuvant-induced arthritis, also significantly improved arthritis score, joint swelling, and local and systemic IL-6 and VEGF levels—both at the gene and protein levels. In this model, ice was more efficient and better tolerated compared to cold gas applications [11].

Our study has a few weaknesses: first, we included patients suffering from a variety of inflammatory rheumatic diseases, inducing some heterogeneity in our study population. This bias was partly corrected by the subgroup analyses we performed (microcrystal-induced arthritides versus RA and SpA), showing globally similar trends of evolution of pro-inflammatory mediators in the synovial fluid after local cryotherapy irrespective of the diagnosis of the inflammatory joint disease, even if the effects seemed to be more pronounced in microcrystal-induced arthritides (Fig. 4, Additional file 2: Figure S2). Furthermore, this population is representative of the patients suffering from acute knee arthritis treated by local cryotherapy in daily clinical practice. And the pro-inflammatory pathways involved in joint inflammation (NF-kB, PG-E2, pro-inflammatory cytokines) are widely shared between these pathologies. Importantly, local cryotherapy—such as corticosteroids and NSAIDs—is an adjuvant anti-inflammatory treatment that is widely used in the acute phase of inflammatory rheumatic diseases. The therapeutic effect of these treatments is mostly not disease-specific (unlike synthetic or biologic disease-modifying anti-rheumatic drugs (DMARDs) for instance), as they target widely shared and pleiotropic pro-inflammatory pathways such as NF-kB and COX-2/PG-E2, as notably demonstrated in our study for local cryotherapy. On the other hand, our results suggest that the molecular effects of local cryotherapy we observed in the synovial fluid of arthritic knees might be generalizable to the four types of inflammatory rheumatic diseases we considered. Further specific studies will be required for each of the four joint inflammatory diseases we included in this preliminary study.

Second, the sample size (*N* = 16) was only calculated in order to demonstrate a significant decrease in synovial IL-6 protein levels, as we found no data in the literature concerning the other cytokine and enzyme level assessment in human synovial fluid. Therefore, we cannot exclude that our study might have been underpowered to detect significant variations in some of the cytokines such as TNF-α or IL-17A. This seems however not so likely, as significant variations could be detected for the other molecular targets.

Thirdly, only 16 patients were treated with cold CO_2_ (versus 31 with ice), as the phase I of the study suggested a superior efficacy of ice, which was therefore the modality chosen in phase II.

Fourthly, some anti-inflammatory drugs (non-steroidal anti-inflammatory drugs (NSAIDs), colchicine) were stopped just 24 h before the start of local cryotherapy. However, it was difficult to stop them for a longer time, as the patients were severely impaired. Furthermore, the half-life of these treatments is short, so the influence of this parameter on our results was likely not major. The results of a clinical study in RA cryotherapy-treated patients suggest that it was important to stop corticosteroids, as the plasma IL-6 levels only decreased in patients with no corticosteroid, with no variation in those under corticosteroids [12]. A corticosteroid intra-joint injection was performed after the second arthrocentesis but had no influence on our results as all the judgment criteria had been gathered at this point.

Fifthly, we included no non-treated arthritic patients as controls in our study. It would have been ethically problematic to leave these painful arthritic patients with no treatment at all except two arthrocenteses for 24 h. Furthermore, no placebo is available for cryotherapy. Therefore, we chose to use contralateral arthritic knees as paired controls when possible. These controls had the advantage to differ only in the absence of cold application compared to the treated knees. Furthermore, our results were widely consistent with those obtained in the study we performed in the murine model of adjuvant-induced arthritis, where non-treated arthritic rats could be used as controls and no concomitant anti-inflammatory drug was used [11].

Sixthly, despite randomization, the CO_2_-treated patients were significantly younger than the ice-treated patients. This difference had no influence on our results in multivariate models.

Seventhly, even if our results suggest that local cryotherapy might reduce cytokine levels in the synovial fluid—at least partly—through NF-kB and PG-E2 pathway inhibition (in the same way as mild tissue hypothermia anti-inflammatory effects that were already described in other pathologies), a more in-depth assessment of the underlying molecular mechanisms was not possible in this preliminary study, due to the limited amounts of synovial fluid. Further studies in animal models or cell culture experiments are now required in order to confirm and better characterize these molecular mechanisms.

Our study also has some strengths: the synovial fluid was always gathered at the same hour (9 a.m.) in order to avoid circadian variations in the cytokine and enzyme levels. In the same study, we could compare ice and cold CO_2_ and evaluate the effects of local cryotherapy in several inflammatory joint diseases. We could also use paired controls (contralateral non-treated knees), which had never been possible in the previous cryotherapy clinical studies. Furthermore, we gained insight into the potential underlying mechanisms of local cryotherapy anti-inflammatory effects.

## Conclusions

In summary, we demonstrated for the first time that local ice cryotherapy decreases synovial IL-6, IL-1β, and VEGF in human knee arthritis, potentially through NF-kB and PG-E2 pathway inhibition.

## Additional files

Additional file 1: **Figure S1.** Inter-class effect size on synovial PG-E2 levels of local ice compared to contralateral non-treated knees. An inter-class effect size (weighted mean difference with 95% CI) was calculated between PG-E2 level evolution (before/after treatment) in ice-treated knees versus corresponding contralateral non-treated knees (*N* = 12) using R® software (meta® and rmeta® packages). The result is expressed in pg/mL. (GIF 5 kb)

Additional file 2: **Figure S2.** Pro-inflammatory mediator evolution after 2 cold applications in the synovial fluid in the non-microcrystal-induced arthritis (RA + SpA) subgroup (*N* = 19). The synovial fluid of all cryotherapy-treated patients suffering from RA or SpA (*N* = 19 including 12 ice-treated patients) was analyzed just before the first cold application, then 24 h later (after 2 cold application). Synovial IL-6 (A), IL-1β (B), TNF-α (C), IL-17A (D), and VEGF (E) assessed by multiplex flow cytometry (CBA® BD Bioscience, Franklin Lakes, NJ, USA); NF-KB-p65 (F)/NF-kB-p65-P (G) assessed by ELISA (85-86083-11®, Thermofisher, Waltham, MA, USA); and PG-E2 (H) assessed by ELISA (KGE004B®, Bio-Techne, Minneapolis, MN, USA) levels were all measured at 9 a.m. (just before the first cold application, then 24 h later). Data are presented as means ± SEM. Paired Wilcoxon-Mann-Whitney tests were performed. *p < 0.05, **p < 0.01, ***p < 0.001. H0: first evaluation (9 a.m., before the first cold application); H24: second evaluation (9 a.m., 24 h later, after the 2 cold applications); RA + SpA: patients suffering from rheumatoid arthritis or spondyloarthritis. Missing values were due to the fact that some cytokines and mediators could not be detected—or with out-of-range values—in some synovial fluid samples before and/or after cold applications (IL-6: *N* = 4 (1 at H0, 1 at H24, 2 at both H0 and H24), *N* = 3 in ice-treated patients (1 at H0, 2 at both H0 and H24); IL-1β: *N* = 1 CO_2_-treated patient (both at H0 and H24); TNF-α: *N* = 0; IL-17A: *N* = 0; VEGF: *N* = 0; NFkB-p65: *N* = 3 (2 at H24, 1 at both H0 and H24), ice-treated patients *N* = 2 (2 at H24); NFkB-p65P: *N* = 3 (2 at H24, 1 at both H0 and H24), ice-treated patients *N* = 2 (2 at H24); PG-E2: *N* = 3 (2 at H24, 1 at both H0 and H24), ice-treated patients *N* = 2 (2 at H24)). (TIF 440 kb)

Additional file 3: **Figure S3.** Correlations between NF-kB synovial protein level evolution, IL-6, and skin temperature drops. Cytokine level variations before/after treatment from all treatment groups (ice, cold CO_2_; *N* = 47 patients) were pooled, and correlation tests were performed using Pearson’s coefficients in order to assess the parameters associated with these cytokine level evolutions. Missing values were due to the fact that some cytokines and mediators could not be detected—or with out-of-range values—in some synovial fluid samples before and/or after cold applications (IL-6: *N* = 4 (1 at H0, 1 at H24, 2 at both H0 and H24); VEGF: *N* = 0; NFkB-p65: *N* = 9 (3 at H0, 4 at H24, 2 at both H0 and H24); NFkB-p65P: *N* = 9 (3 at H0, 4 at H24, 2 at both H0 and H24)). (TIF 203 kb)

## Data Availability

The datasets used and/or analyzed during the current study are available from the corresponding author on reasonable request.

## Abbreviations

COX-2: Cyclo-oxygenase 2
CPDD: Calcium pyrophosphate deposition disease
DMARD: Disease-modifying anti-rheumatic drug
IL-6: Interleukin 6
NF-kB: Nuclear factor kappa B
NF-kB-p65(-P): (Phosphorylated) nuclear factor kappa B p65
NSAIDs: Non-steroidal anti-inflammatory drugs
Pain VAS: Pain visual analog scale
PG-E2: Prostaglandin E2
RA: Rheumatoid arthritis
SpA: Spondyloarthritis
TNF-α: Tumor necrosis factor alpha
VEGF: Vascular endothelial growth factor
