# Supplementary figures and images for: Local ice cryotherapy decreases synovial interleukin 6, interleukin 1β, vascular endothelial growth factor, prostaglandin-E2, and nuclear factor kappa B p65 in human knee arthritis: a controlled study
Source: Arthritis Res Ther. 2019 Jul 30;21:180. doi: 10.1186/s13075-019-1965-0 (PMC6668066; doi:10.1186/s13075-019-1965-0)

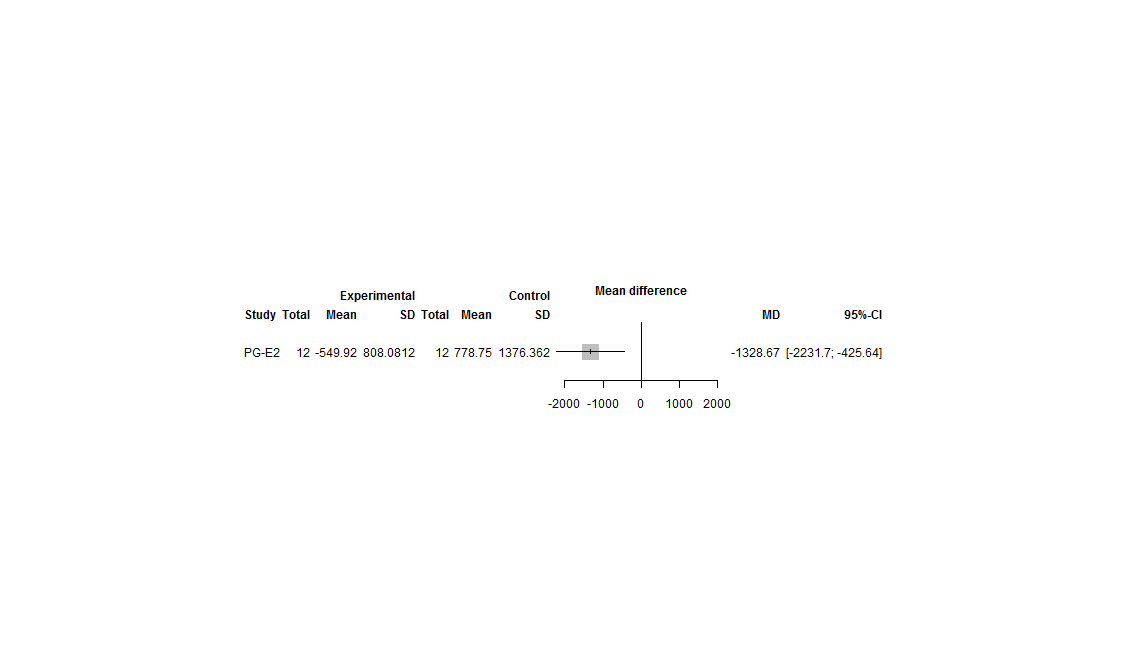

Supplement: Supplementary file 1 — Figure S1. Inter-class effect size on synovial PG-E2 levels of local ice compared to contralateral non-treated knees. An inter-class effect size (weighted mean difference with 95% CI) was calculated between PG-E2 level evolution (before/after treatment) in ice-treated knees versus corresponding contralateral non-treated knees (N = 12) using R® software (meta® and rmeta® packages). The result is expressed in pg/mL. (GIF 5 kb) [file 13075_2019_1965_MOESM1_ESM.gif]

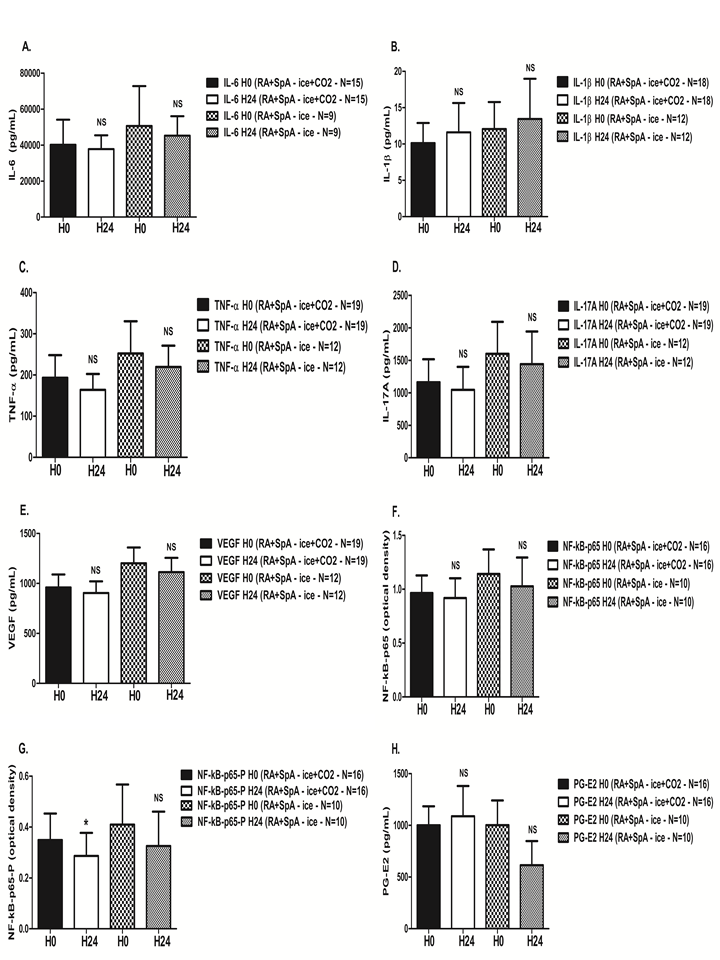

Supplement: Supplementary file 2 — Figure S2. Pro-inflammatory mediator evolution after 2 cold applications in the synovial fluid in the non-microcrystal-induced arthritis (RA + SpA) subgroup (N = 19). The synovial fluid of all cryotherapy-treated patients suffering from RA or SpA (N = 19 including 12 ice-treated patients) was analyzed just before the first cold application, then 24 h later (after 2 cold application). Synovial IL-6 (A), IL-1β (B), TNF-α (C), IL-17A (D), and VEGF (E) assessed by multiplex flow cytometry (CBA® BD Bioscience, Franklin Lakes, NJ, USA); NF-KB-p65 (F)/NF-kB-p65-P (G) assessed by ELISA (85-86083-11®, Thermofisher, Waltham, MA, USA); and PG-E2 (H) assessed by ELISA (KGE004B®, Bio-Techne, Minneapolis, MN, USA) levels were all measured at 9 a.m. (just before the first cold application, then 24 h later). Data are presented as means ± SEM. Paired Wilcoxon-Mann-Whitney tests were performed. *p < 0.05, **p < 0.01, ***p < 0.001. H0: first evaluation (9 a.m., before the first cold application); H24: second evaluation (9 a.m., 24 h later, after the 2 cold applications); RA + SpA: patients suffering from rheumatoid arthritis or spondyloarthritis. Missing values were due to the fact that some cytokines and mediators could not be detected—or with out-of-range values—in some synovial fluid samples before and/or after cold applications (IL-6: N = 4 (1 at H0, 1 at H24, 2 at both H0 and H24), N = 3 in ice-treated patients (1 at H0, 2 at both H0 and H24); IL-1β: N = 1 CO2-treated patient (both at H0 and H24); TNF-α: N = 0; IL-17A: N = 0; VEGF: N = 0; NFkB-p65: N = 3 (2 at H24, 1 at both H0 and H24), ice-treated patients N = 2 (2 at H24); NFkB-p65P: N = 3 (2 at H24, 1 at both H0 and H24), ice-treated patients N = 2 (2 at H24); PG-E2: N = 3 (2 at H24, 1 at both H0 and H24), ice-treated patients N = 2 (2 at H24)). (TIF 440 kb) [file 13075_2019_1965_MOESM2_ESM.tif]

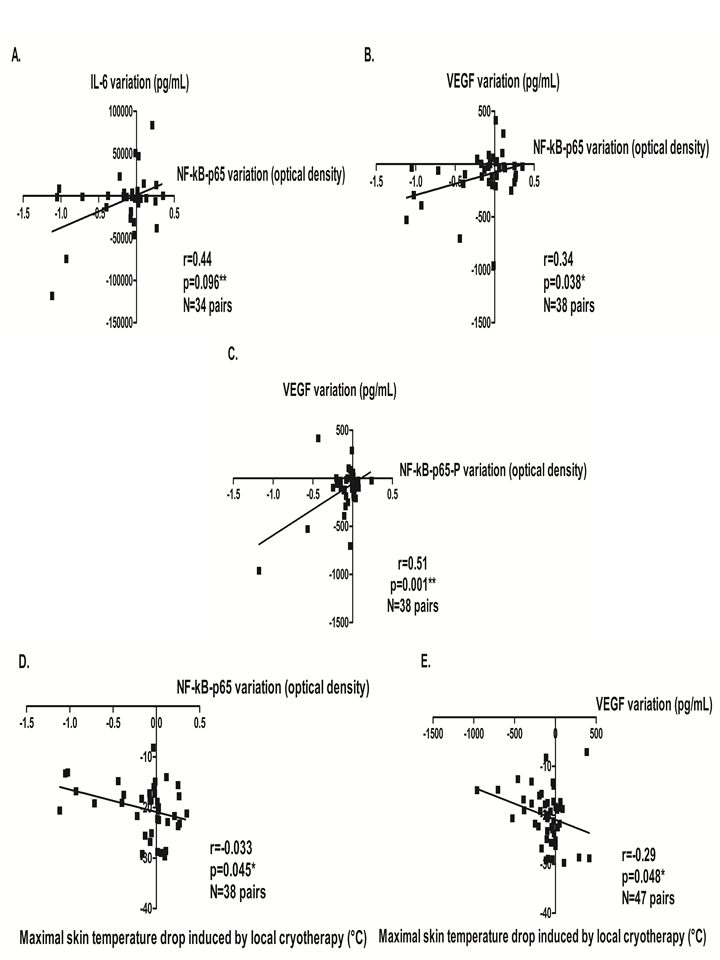

Supplement: Supplementary file 3 — Figure S3. Correlations between NF-kB synovial protein level evolution, IL-6, and skin temperature drops. Cytokine level variations before/after treatment from all treatment groups (ice, cold CO2; N = 47 patients) were pooled, and correlation tests were performed using Pearson’s coefficients in order to assess the parameters associated with these cytokine level evolutions. Missing values were due to the fact that some cytokines and mediators could not be detected—or with out-of-range values—in some synovial fluid samples before and/or after cold applications (IL-6: N = 4 (1 at H0, 1 at H24, 2 at both H0 and H24); VEGF: N = 0; NFkB-p65: N = 9 (3 at H0, 4 at H24, 2 at both H0 and H24); NFkB-p65P: N = 9 (3 at H0, 4 at H24, 2 at both H0 and H24)). (TIF 203 kb) [file 13075_2019_1965_MOESM3_ESM.tif]
